# Study preregistration: an early example and analysis

**DOI:** 10.7717/peerj.21360

**Published:** 2026-05-25

**Authors:** Richard Wiseman, Caroline Watt

**Affiliations:** 1 Department of Psychology, Sport and Geography, University of Hertfordshire, Hatfield, Hertfordshire, United Kingdom; 2 School of Philosophy, Psychology and Language Sciences, University of Edinburgh, Edinburgh, United Kingdom

**Keywords:** Psychology, Preregistration, Methods, Meta-science, Parapsychology, Study registration

## Abstract

Preregistration is commonly seen as an effective way of reducing questionable research practices within psychology. This procedure happens prior to data collection/analysis and usually involves either sending in a research plan to a study registry or submitting a registered report. A prototype of registered reports operated within parapsychology long before mainstream psychology, and recent research showed that these reports contained fewer significant results than traditional journal articles. The current study builds on this work by examining the efficacy of another early preregistration procedure within parapsychology, namely the Koestler Parapsychology Unit’s Study Registry (KPU SR). This initiative began in 2012, has attracted almost a hundred submissions, and continues to operate. The outcomes of studies preregistered on the KPU SR were compared with those of non-preregistered parapsychology studies published in journals during the same time period. In line with previous work within mainstream psychology, the preregistered studies contained fewer significant results (16%) than non-preregistered experiments (46.6%). The implications of these findings for preregistration are discussed along with suggestions for future research.

## Introduction

Bem (2011) published a series of studies in the *Journal of Personality and Social Psychology* that appeared to support the existence of precognition (the hypothesised ability to see into the future). The high-profile nature of the journal and controversial subject matter resulted in Bem’s work attracting considerable critical scrutiny, with commentators often arguing that the findings were due to various ‘questionable research practices’ (QRPs), including hypothesizing after the results are known, multiple analyses, data selection and publication bias (Alcock, 2011; Francis, 2012; Schimmack, 2012).

Wagenmakers et al. (2011) highlighted a key issue, namely that the methodological similarities between Bem’s work and many mainstream psychology studies implied that a significant amount of the latter work also involved QRPs and thus ran the risk of producing equally spurious findings. Several strands of evidence supported this argument, including: surveys showing that experimenters frequently engage in QRPs (Agnoli et al., 2017; John, Loewenstein & Prelec, 2012; Makel et al., 2021); papers containing an implausibly large number of significant results (Fanelli, 2010); researchers struggling to replicate well-known psychological findings (OpenScience Collaboration, 2015); and evidence of publication bias (Franco, Malhotra & Simonovits, 2014; Moniz, Druckman & Freese, 2025).

Whereas earlier concerns about the methodological and statistical integrity of psychology studies had received relatively scant attention (*e.g.*, Walster & Cleary, 1970; Mahoney, 1977; Newcombe, 1987), this new wave of criticism attracted far more interest (Lakens, 2025). Much of the resulting discussion centred on how QRPs could be minimised by experimenters preregistering key aspects of a study, (including the hypotheses, method, and analyses) prior to data collection/analysis by sending a research plan to a study registry, or submitting a registered report to a journal (Nosek et al., 2018; Wagenmakers et al., 2012; Van’t Veer & Giner-Sorolla, 2016). Both study registries and registered reports have now been operating within psychology for over a decade, with some of the first preregistered studies appearing on the Open Science Framework SR in 2012 and *Cortex* becoming the first psychology journal to accept registered reports in 2013 (Chambers, 2017).

As psychology’s database of preregistered studies accumulates, researchers have begun to examine whether preregistration reduces spurious positive findings. Schäfer & Schwarz (2019) compared the first hypothesis tested in preregistered studies with non-preregistered articles randomly sampled from the psychological literature. 64% of the preregistered work reported a positive effect *versus* 79% of the non-preregistered studies. Scheel, Schijen & Lakens (2021) employed a similar procedure and found that 44% of preregistered studies contained a statistically significant result compared to 96% of non-preregistered studies. Toth et al. (2021) collated all formal analyses reported in preregistered studies and compared them to non-preregistered articles appearing in the same journal issues as the preregistered work. They found that 48% of preregistered studies reported a significant effect *versus* 66% of non-preregistered work. All three projects employed preregistered studies drawn from registered reports, or a mix of registered reports and work submitted to study registries. In contrast, Van den Akker et al. (2024) compared work that had only been preregistered on a study registry (either because it had been entered into a competition to promote preregistration or had been awarded a preregistration badge) with non-preregistered studies linked to each preregistered publication *via* the Web of Science. Considering all formal analyses in each paper, the percentage of positive findings in each group was around 68% and the difference between the groups was nonsignificant.

Similar analyses have been undertaken within medicine, wherein researchers are encouraged to preregister their work using study registries that involve a considerable amount of scrutiny. The findings from these analyses are mixed but tend to show that preregistration is associated with smaller effect sizes. For example, Odutayo et al. (2017) found no difference in the outcomes of registered and non-registered clinical trials. In contrast, Kaplan & Irvin (2015) reported a drop in the effect size of studies evaluating treatments for cardiovascular disease once researchers were required to preregister key aspects of their studies. Focusing on studies included in Cochrane reviews, Dechartres et al. (2016) discovered that unregistered trials showed larger effects than registered trials. Papageorgiou et al. (2018) found the same pattern among studies examining the effects of orthodontic clinical interventions.

Parapsychology is usually defined as the scientific investigation of alleged psychic ability, and includes studies examining the apparent awareness of information not gained through the traditional senses (commonly known as extra-sensory perception or ESP) and the alleged mental influence of a physical or biological system (psychokinesis or PK). This controversial subject-matter has attracted considerable debate between parapsychologists and critics that, historically, has tended to focus on methodological issues specific to parapsychology, such as adequacy of safeguards against sensory leakage and randomisation procedures (*e.g.*, Hyman, 1985; Palmer, 1987). However, parapsychologists have also long discussed how the replicability of their work might be affected by QRPs. For instance, when assessing a large number of ESP studies, Rhine et al. (1940) discussed several QRPs, including data selection and multiple analyses. Later, Wiklund (1977) showed how it was possible to use *post-hoc* analyses to generate illusory effects, thus foreshadowing similar work in mainstream psychology by over 30 years (Simmons, Nelson & Simonsohn, 2011). Similarly, in the mid-1970s, parapsychologists fiercely debated issues surrounding publication bias (Rhine, 1975; Johnson, 1976) and this led to the Editors of the now defunct *European Journal of Parapsychology* (*EJP*) establishing a prototype of registered reports that ran from 1976 to 1992. This work pre-dates similar initiatives in mainstream psychology, and Wiseman, Watt & Kornbrot (2019) recently showed that the scheme was impactful, with 8.4% of the registered reports having significant findings compared to 28.4% of the non-registered reports. Parapsychology may therefore serve as a useful model for psychologists grappling with replication issues.

Parapsychology’s first study registry was launched in 2010, a few years before similar work in mainstream psychology (Notes from Two Scientific Psychologists, 2010). This small-scale initiative was designed to collate attempts to replicate Bem’s precognition work and only attracted a handful of studies. A far more comprehensive and wide-ranging study registry was launched at Edinburgh University’s Koestler Parapsychology Unit (KPU) by Jim Kennedy and Caroline Watt in 2012 and continues to attract submissions (Watt & Kennedy, 2015). This is the only study registry specialising in parapsychological research, can be used to preregister any study testing hypothesised psychic ability, and currently houses research plans for almost 100 studies. The KPU study registry requires researchers to state whether a study is exploratory or confirmatory. Although the reporting requirements for exploratory work are relatively light, researchers submitting confirmatory studies are required to provide a substantial amount of additional information, particularly concerning analysis decisions that could influence results (*e.g.*, whether statistical tests are one- or two-sided, rules for excluding data, and the criterion for acceptable evidence). Confirmatory submissions are rigorously reviewed, with ambiguities or omissions being resolved before acceptance. Once accepted, the full registration document is posted on a public website to which only the administrators have editorial access. This document cannot be changed or withdrawn after testing has commenced. Periodically researchers are invited to provide a brief report of their results, or a published journal article if available, and this information is also uploaded onto the public website. Work submitted to a mainstream psychology study registry usually receives a relatively low level of scrutiny and the process tends to be self-policing. In contrast, the research plans sent to the KPU study registry are subjected to a considerable amount of critical attention. To separate these two approaches, the KPU approach will be referred to as a ‘managed study registry.’

Complementing Wiseman, Watt & Kornbrot (2019)’s analysis of the impact of registered reports in parapsychology, the current work compares the outcomes of studies that were preregistered on the KPU managed study registry with non-preregistered parapsychological studies. Previous research into the impact of preregistration in psychology has selected a subset of preregistered and non-preregistered papers from the vast psychology literature. However, the relatively modest scale of parapsychological research makes it possible to utilise a more comprehensive comparison, namely comparing all studies in parapsychology’s only active study registry with every eligible study published in the same journals that published the preregistered findings. In line with previous research, it was expected that the preregistered studies would be significantly less likely than the non-preregistered studies to contain findings supporting the study hypotheses. An additional analysis explored whether any effect might be moderated by the two groups of studies examining different topics (ESP *vs* PK). All analyses were exploratory, and all data exclusions and measures have been reported.

## Method

### Design

This study utilised a retrospective archival design.

### Dataset and coding

The dataset contained studies that experimentally examined the existence of psychic ability and comprised of two groups. The preregistered group consisted of confirmatory studies submitted to the KPU managed study registry between its inception in November 2012 and the cut-off date (1st November 2025). For this group, the number of confirmatory hypotheses was coded from the study preregistration document and the number of supported hypotheses was coded from the results posted to the KPU managed study registry or from the published paper where available. The non-preregistered group consisted of non-preregistered papers that were published in the nine journals that published the preregistered studies within the same time-period (*The Journal of the Society for Psychical Research*; *Journal of Parapsychology*; *Journal of Scientific Exploration*; *Journal of Anomalous Experience and Cognition*; *Journal of Consciousness Studies*; *Psychology of Consciousness: Theory, Research and Practice*; *Explore: The Journal of Science and Healing*; *F1000Research*; *Neuroquantology*). All the work published in the first seven journals was assessed for possible inclusion and studies in the latter two online journals were identified using a comprehensive list of search terms (parapsychology, paranormal, psychic, remote viewing, random number generator, non-local, precognition, anomalous cognition, ganzfeld, interaction at a distance). To help ensure that the non-preregistered group studies matched the preregistered group studies, any experiments or hypotheses described as pilot or exploratory were excluded. This decision was made prior to coding. As with Wiseman, Watt & Kornbrot (2019), studies were excluded if they reported a meta-analysis, an artefact that the author(s) believed undermined the experiment, or didn’t provide a statistical evaluation of the study outcome.

Each study was assigned an identification number (ExperimentID) and the following three variables were coded:

***H:*** Total number of formal hypotheses tested.

***S:*** Total number of formal hypotheses supported. As with Wiseman, Watt & Kornbrot (2019), nearly all papers employed *p*-values to assess hypotheses (usually using *p* < .05 as the criterion for significance) and thus this metric was adopted. On the few occasions where Bayesian analyses were employed, the result was seen as supportive when Bayes factors (BFs) were either smaller than 1/3 or greater than 3.

**T:** Whether the primary topic of the study was to test for ESP or PK.

The two authors independently coded each paper and resolved any disagreement *via* discussion. These disagreements occurred a relatively small number of times (approximately 15% of the papers) and tended to focus on identifying the number of formal hypotheses involved in each study.

## Results

The KPU managed study registry contained 45 exploratory and 53 confirmatory studies. The preregistered group consisted of the 38 confirmatory studies that had posted results. Some confirmatory studies involved more than one experiment, and thus the final dataset consisted of 42 experiments that tested 100 hypotheses. The non-preregistered group contained 71 papers that described 93 experiments that tested 204 hypotheses.

**Number of hypotheses supported.** A total of 16% of the hypotheses in the preregistered group were significant (16/100; 95% CI [9.43%–24.68%]) compared to 46.6% of those in the non-preregistered group (95/204; 95% CI [39.57%–53.76%]). A 2 × 2 contingency analysis comparing the groups was highly significant (Chi-square = 25.75, *df* = 1, *p* <  .0001, Phi = .30).

In addition, to address any issues over possible lack of independence of hypotheses within experiments, we carried out a *post hoc* analysis using each experiment as the unit of analysis and comparing those that had at least one hypothesis supported with those that did not. 33% of the experiments in the preregistered group had at least one supported hypothesis (14/42; 95% CI [19.08%–47.59%]) compared to 63% of those in the non-preregistered group (59/93; 95% CI [53.65%–73.23%]). A 2 × 2 contingency analysis comparing these groups was highly significant (Chi-square = 10.56, *df* = 1, *p* <  .001, Phi = .28).

The *p*-values associated with the above analyses are associated with a BF > 3 (usually adopted as support for the hypothesis by Bayesians).

**Topic.** 78.9% of the papers in the preregistered group reported ESP experiments (30/38 CI [62.68%–90.45%]) compared to 67.6% of those in the non-preregistered group (48/71 CI [55.54%–78.24%]). A 2x2 contingency analysis comparing these groups was not significant (Chi-square = 1.06, *df* = 1, *p* =. 31, Phi = .12).

## Discussion

For over a decade, psychologists have attempted to reduce potential QRPs by pre-registering their research on a study registry or in a registered report. Parapsychologists conducted early work into these topics, including setting up a prototype registered report system before mainstream psychology that was effective in reducing spurious positive findings (Wiseman, Watt & Kornbrot, 2019). The KPU managed study registry was initiated around the same time as study registration began in mainstream psychology, has consistently attracted submissions, and continues to operate as parapsychology’s only study registry. Its distinctive features include a thorough review of submissions and the irreversibly public posting of study plans (Watt & Kennedy, 2015). The current study examines how preregistering work on the KPU managed study registry impacts study outcome by comparing the results of these submissions with non-preregistered papers published in the same time frame.

The main analysis showed that significantly fewer hypotheses were supported in studies that were preregistered on the KPU managed study registry (16%) compared to non-preregistered work (46.6%). This pattern is in line with similar analyses within mainstream psychology (*e.g.*, Schäfer & Schwarz, 2019; Scheel, Schijen & Lakens, 2021; Toth et al., 2021) and provides further evidence that pre-registration reduces spurious positive findings. Indeed, this finding strengthens confidence in this notion as the current work was undertaken within an area that wasn’t part of the previous analyses, and involved a more complete and comprehensive set of both preregistered (all studies in parapsychology’s only study registry *versus* selected psychology registered reports or study registries) and non-preregistered research (all eligible studies in the same journals as the preregistered studies *versus* a selection of publications from the psychological literature). However, as with all research of this type, it’s important to exercise caution as the studies were not randomly allocated to the preregistered group and non-preregistered group, and so could vary on other factors. It is reassuring to know that in the current work, the two groups of studies did not differ in terms of the general topic under investigation (ESP or PK), but it is still possible they could differ on other factors, such as statistical power, methodological quality, and opportunities for experimenter fraud. Given that this is the case, the findings should be seen as indicative rather than definitive.

This finding also helps to extend our understanding of the relationship between QRPs, registered reports and study registries. The previous analyses of preregistration and outcome in psychology studies have employed a mixture of registered reports and work submitted to study registries. However, the current investigation focussed exclusively on work submitted to the KPU managed study registry. As noted in the Introduction, the only previous study to have analysed purely work that had been submitted to a study registry (Van den Akker et al., 2024) failed to find any difference in the study outcomes of preregistered and non-preregistered studies. Although this finding is open to several interpretations, it’s possible that study registries are not as effective as registered reports at reducing QRPs because they tend to involve a lower level of scrutiny, and/or it’s easier for researchers to hide a study on a study registry than to retract a registered report. The current analysis supports this notion because the scope and scale of psychology study registries means that submitting work is a self-directed and self-policed process rather than involving peer review. In contrast, the KPU managed study registry involves high levels of both scrutiny and transparency with the plans for confirmatory studies receiving a considerable amount of critical attention. Indeed, when describing the operation of the KPU managed study registry, the administrators noted that revision was required for almost every submission (Watt & Kennedy, 2015). In addition, the KPU managed study registry is public, only the registry administrators have editorial access, and researchers are unable to hide submissions once they are accepted. Together, this supports the assertion that study registries only reduce QRPs when they involve a significant amount of scrutiny and transparency. Such peer review would be problematic in large-scale psychology study registries because of the scope and volume of work, but one way forward would be for those working in specific areas of psychology to create and run more small-scale and bespoke study registries. Furthermore, preregistration is not a panacea. Within clinical trials (an early adopter of preregistration) around one third of preregistered trials have a discrepancy between the preregistered and the published primary outcome measure (Jones et al., 2015; see also Goldacre et al., 2019; Claesen et al., 2021). The present study did not investigate this issue, however there is little reason to expect psychology and parapsychology to do better, and this area merits further investigation.

Wiseman, Watt & Kornbrot (2019)’s reported that only 8.4% of hypotheses were supported in registered reports submitted to a parapsychology journal, and this figure did not provide strong support for the existence of psychic ability. The present study found that around 16% of hypotheses were supported in the preregistered studies submitted to the KPU managed study registry. However, when seeking to interpret the evidential nature of this finding, it is important to remember that although preregistration helps to reduce QRPs, the experiments in the preregistered group may contain other methodological issues, such as sensory leakage and poor randomisation (Milton & Wiseman, 1997). In addition, of the 53 confirmatory studies posted to the KPU managed study registry prior to the cut-off, eight are yet to return results and five were reported as discontinued. Thus, our database of preregistered studies may itself be affected by filedrawer effects.

The percentage of significant hypotheses reported in both the preregistered and non-preregistered parapsychological work (current analysis 16% & 47%; Wiseman, Watt & Kornbrot (2019) 8% & 28%) is considerably lower than that reported in similar meta-scientific investigations within mainstream psychology (Schäfer & Schwarz, 2019 64% & 79%; Scheel, Schijen & Lakens (2021) 44% & 96%; Toth et al. (2021) 48% & 66%; Van den Akker et al., 2024 68%). This could be due to the fragile, and possibly non-existent, nature of the effects investigated within parapsychology (Reber & Alcock, 2020). Alternatively, it could be that parapsychology’s long history of trying to eliminate QRPs, especially those concerned with publication bias, may have resulted in increased methodological and statistical rigour. Future work on parapsychological findings could examine this phenomenon, including mirroring previous work within mainstream psychology that has investigated the relationship between study outcome and speed of publication (Ioannidis, 1998), and whether publication bias is driven by researchers being more likely to submit papers reporting significant results and/or reviewers and editors evaluating them in a more favourable light (Mahoney, 1977).

Finally, a significant amount of previous work suggests that psychology may benefit from greater insight into some aspects of parapsychology. For example, Wiseman & Watt (2025) noted that pioneering parapsychologists have uncovered important psychological phenomena, including the unreliability of eyewitness testimony, inattentional blindness, and hindsight bias. Similarly, on a methodological level, parapsychologists have helped to pioneer the development of many now-standard methodological procedures, including randomisation (Hacking, 1988) and blind procedures (Kaptchuk, 1998). In terms of meta-science, Wiseman, Watt & Kornbrot (2019) noted that parapsychologists instigated prototype registered reports decades before the practice was adopted within mainstream psychology. The current work is another illustration of how parapsychology is relevant to mainstream psychology (Watt, 2005; Wiseman & Watt, 2025). Given that this is the case, it is hoped that the type of work reported here will help to increase psychologists’ awareness of this small but important area of research.

## Conclusions

Previous research has shown that parapsychologists pioneered a prototype of registered reports and that this helped to reduce positive but illusory effects. The current paper complements this work by investigating the impact of the only study registry for parapsychological experiments. The KPU managed study registry was launched around the time of similar efforts within mainstream psychology, and the present study found that confirmatory preregistered studies contained significantly fewer significant hypotheses than non-preregistered journal articles. These findings add to, and extend, existing literature on meta-science in mainstream psychology and underscore the importance of high levels of scrutiny and transparency in supporting the efficacy of study registries.

##  Supplemental Information

## Additional Information and Declarations

### Data Availability

The following information was supplied regarding data availability: The raw data is available in the Supplemental Files.
